# Chemical constituents, antibacterial and antioxidant properties of the essential oil flower of *Tagetes minuta* grown in Cala community Eastern Cape, South Africa

**DOI:** 10.1186/s12906-017-1861-6

**Published:** 2017-07-05

**Authors:** Aboi Igwaran, Benson Chucks Iweriebor, Sunday Ofuzim Okoh, Uchechukwu Uchechukwu Nwodo, Larry Chikwelu Obi, Anthony Ifeanyi Okoh

**Affiliations:** 1 SAMRC Microbial Water Quality Monitoring Centre, University of Fort Hare, Alice, 5700 South Africa; 2 Applied and Environmental Microbiology Research Group (AEMREG), Department of Biochemistry and Microbiology, University of Fort Hare, Private Bag X1314, Alice, Eastern Cape Province, 5700 South Africa; 3 Academic and Research Division, University of Fort Hare, Private Bag X1314, Alice, Eastern Cape Province, 5700 South Africa

**Keywords:** Antibacterial, Antioxidant, *Tegates minuta*, Vitamin C

## Abstract

**Background:**

*Tagetes minuta* has a long record of human use for the treatment of stomach and intestinal diseases. Most drugs used for diseases treatment are less efficacious with side effects and this brought the search for new treatment regimens mainly from medicinal plants.

**Method:**

The essential oil (EO) was extracted by Clevenger’s-type apparatus and its chemical composition, antioxidant and antibacterial properties were determined by GC-MS, spectrophotometric and broth dilution methods respectively. *S. uberis*, *E. cloacae*, *S. aureus*, *M. smegmatis*, *L. ivanovii*, *Vibrio* spp. and *E. coli* bacteria strains were used as test bacteria.

**Results:**

GC-MS analysis revealed 98 compounds in the EO flower of *T. minuta* and β-Ocimene (14. 40%) was the major chemical constituents. The EO exhibited highest inhibitory effect against DPPH radical, followed by its effect on ABTS, while LP radical showed the least sensitivity with IC_50_ values of 2.45 mg/mL, 2.76 mg/mL and 3.23 mg/mL respectively. The EO showed antibacterial activities against all test organisms with MIC value for *S. aureus*, *M. smegatis* and *S. uberis* at 0.125 mg/mL and for *L. ivanovii*, *Vibrio* spp., *E. cloacae* and *E. coli* at 0.06 mg/mL. The EO showed MBC against *E. cloacae* and *E. coli* at 0.06 mg/mL at 0.5 mg/mL for *S. uberis* and 0.125 mg/mL for *Vibrio* spp.

**Conclusion:**

Findings from this study suggest that the EO of *T. minuta* flower may be a useful candidate in the search for lead constituents for the synthesis of new potent antibacterial and antioxidant agent.

## Background

Free radicals instigate oxidative damage and when this free radical in the body system is above the capacity of antioxidant, it results in oxidative stress which is implicated in many human diseases [1]. Antioxidant is substance that helps to prevent oxidative damage when found in small amounts and is equivalent to an oxidizable substrate. Antioxidants aid in averting of diseases by scavenging radicals including lipid peroxyl (LP •), superoxide (O_2_ •), nitric oxide (NO •) and hydroxyl (HO •) formed during metabolic activities [2]. The need for natural antioxidants is becoming imperative due to numerous health risk associated with synthetic antioxidants [3]. Several in vitro assays such as DPPH, ABTS, lipid peroxyl and ferric reducing ability of plasma (FRAP) radicals have been used in evaluating antioxidants capacity of plants extracts which are designed based on quenching stable free radicals [4]. Plants used traditionally are known to produce a wide range of compounds with therapeutic properties such as antioxidant, antibacterial, gastroprotective effects amongst others [5].

Study has shown that the continuous use of synthetic antibiotics for a long period is one of the causes of bacterial resistant [6]. The treatment failures associated with multidrug-resistant bacterial strains including methicillin-resistant *Staphylococcus aureus* (MRSA), vancomycin-resistant *Staphylococcus aureus* (VRSA), vancomycin-resistant *Enterococcus faecalis, Vibrio* Spp*.* and *Escherichia coli* have become a worldwide concern [7], which has heightened the search for alternative therapeutic agents [8]. The use of herbal medicine for the treatment of diseases has been stated by World Health Organization (WHO) and presently a lot of persons use herbal medicine for treatment of diverse disease [9, 10]. Plant extracts provide boundless opportunities for such option as well as in discovery of new drugs because of availability of potent chemical components present in their extracts [11, 12]. The active compound found in medicinal plants that exhibit therapeutic activities against pathogens have little or no side effects on the host cells [13]. Phytochemical compounds present in the essential oil (EO) of plants are diverse, complicated and are known to contain active antibacterial property [14] and antioxidant properties [15]. Essential oils from medicinal plants contain naturally occurring antimicrobial compounds which have been shown to be effective in limiting the growth and survival of many pathogens [16]. These essential oils from plant such as *Eucalyptus*, *Pogostemon cablin* and tea tree have been reported to exhibit antimicrobial potential [17], and this make EO appropriate option to synthetic antibiotics [18].

*Tagetes minuta* is commonly called wild marigold also known as Mexican marigold of the family *Asteraceae* [19], and it belongs to one of the 56 species of *Tagetes* [20]. *T. minuta* is found in many countries such as Argentina and South America [21], including South Africa. *T. minuta* is commonly called nnkayo by the Xkosa people, a tribe in the Easter Cape Province, South Africa. *T. minuta* has been reported to have a long record of human use for insect repellent, treatment of stomach and intestinal diseases [22]. There has been heightened interest of late in plant-based natural products that have the ability to reduce free radicals formation and treating infections caused by pathogenic microorganisms. There is however scarce information on antibacterial and antioxidant properties as well as the chemical constituents of the essential oil flower of *T. minuta* grown in Cala community, Eastern Cape Province, South Africa that is claimed to be potent against many infections. This study therefore aimed to evaluate antibacterial, antioxidant properties and the chemical constituents of the essential oil of *T. minuta* flower grown in Cala community South Africa.

## Methods

### Plant material

Fresh flowers of *T. minuta* were collected from Cala community located in Sakhisizwe Local Municipality Eastern Cape Province, South Africa with geographical coordinates of 31° 33′ 0″ South, and 27° 36′ 0″ East [23]. Taxonomical identification of the plant was confirmed at.

Selmer Schonland Herbarium, Albany Museum Grahams town with Voucher No. BM01–040/2007 and the voucher specimen was deposited. Prior to essential oil extraction, plant material was rinsed with distilled water and shade dried on foil paper in the laboratory at ambient temperature for 6 days and thereafter, *T. minuta* flower was pulverized in a blending machine (Polymix PX-MFC90 D, Lasec/SA).

### Extraction of essential oil

The essential oil obtained was extracted from the powdered flower (376.86 g) for 3 h with a modified hydro-distillation Clevenger’s-type apparatus as described by Omoruyi et al., [24]. The hydro-distillation experiment was carried out thrice to obtain enough oil for bioactivity assays and the extracted essential oil was dried over anhydrous sodium sulphate, dispensed into tinted vials and stored at 4 °C. The yield of the essential oil was determined in *w*/w% (per gram) of the extracted plant sample.

### Gas chromatography mass spectrometry (GC-MS)

GC-MS analyses of the essential oil was performed on Agilent 5977A MSD and 7890B GC system, Chemetrix (pty) Ltd.; Agilent Technologies, DE (Germany) with a Zebron-5MS column (ZB-5MS 30 m × 0.25 mm × 0.25 um) (5% -phenylmethylpolysiloxane). The following column and temperature conditions used were: GC grade helium at a flow rate of 2 mL/min and splitless 1 mL injections was used. The injector, source and oven temperatures were set at 280 °C, 280 °C and 70 °C, respectively. The ramp settings were set at; 15 °C/min to 120 °C, afterwards 10 °C/min to 180 °C, then 20 °C/min to 270 °C and held for 3 min.

### Chemical reagents

The chemicals used were acetic acid, thiobarbituric acid (TBA), trychloroacetic acid, sodium dodecyl sulphate (SDS), 2, 2-diphenyl-1-picrylhydrazyl (DPPH), 2, 2′-azino-bis-3-ethylbenzthiazoline-6-sulphonic acid (ABTS), ethanol, methanol, ethyl acetate, n-hexane, Iron (II) sulphate (FeSO_4_), sodium hydroxide (NaOH), ascorbic acid and butanol. All chemicals reagents were of analytical quality and were bought from reliable commercial sources.

### Determination of antioxidant property

Antioxidant property of the extracted essential oil of *T. minuta* flower was evaluated in vitro by spectrophotometric method against DPPH, ABTS and lipid peroxyl radicals.

### DPPH radical scavenging assay

DPPH assay was carried out following the modified method of Ajileye et al., [25]. Briefly, the solution of DPPH (0.135 mM) was prepared in methanol and incubated in the dark for 30 min. One hundred microliter of the essential oil or standards (positive control) were prepared in methanol of various concentration ranging from (0.03–0.5 mg/mL), and was added into all the wells of microtiter plate starting from (C-H) in triplicates except for A (A1-A12) and B (B1-B12). Thereafter, 100 μL of 0.135 mM of DPPH solution prepared in methanol was added into the wells from C-H. Absorbance was spectrophotometrically observed at 517 nm and the essential oil ability to lower DPPH to neutral molecule was expressed as percentage inhibition using the formula percentage inhibition = {(Abs _control_ –Abs _sample_)}/ (Abs control) × 100.

### ABTS scavenging assay

ABTS test was determined following the modified method of Kannan et al., [26]. Briefly, stock solutions was prepared by mixing two stock solutions (1:1 *v*/v) ratio of potassium persulfate (2.45 mM) and ABTS (7.0 mM), incubated in a dark cupboard for 720 min at ambient temperature. One microliter of ABTS+ solution was diluted by adding methanol to obtain an absorbance of 0.708 ± 0.002 unites when measured at 734 nm with spectrophotometer. Briefly, 100 μL of the essential oil or standards prepared in methanol of various concentration ranging from 0.03–0.5 mg/mL was added into all the wells of microtiter plate starting from (C-H) in triplicates except for A (A1-A12) and B (B1-B12). Thereafter, 100 μL of ABTS+ solution was then added to all the wells starting from C-H and the solution was incubated for 7 min and absorbance was taken at 734 nm using spectrophotometer. The percentage inhibition was calculated using the formula stated above.

### Lipid peroxidation by TBARS test

The modified method of thiobarbituric acid reactive species (TBARS) assay described by Badmus et al., [27] was adapted to measure the inhibitory effect of the essential oil on lipid peroxidation using egg yolk homogenates as lipid rich source. A volume of 125 μL of 10% of the egg homogenate (in distilled water) was added to various concentrations ranged from 0.03–0.5 mg/mL of the plant extract prepared in methanol. The volume was adjusted to 250 μL with distilled water. Afterward, 12.5 μL of FeSO_4_ (Iron (II) sulphate) was added to the solution and incubated at 25 °C for 30 min. A 375 μL of 10% acetic acid (pH 3.50) adjusted with NaOH. Then, 0.80% of 2-thiobarbituric acid (375 μL) mixed with sodium dodecyl sulphate 1.1% and 2% trichloroacetic acid (12.5 μL) was added in the same micro centrifuge tubes, vortexed and heat at 65 °C for 60 min. After cooling, 975 μL of butanol was added, centrifuged at 3000 rpm for 600 s. The upper organic layer was then aspirated and the absorbance read at 532 nm. Percentage inhibition of lipid by the EO was calculated using the formula previously stated above and all the assays were performed in triplicate.

### Antibacterial test

#### Test organisms

The reference bacteria strains used are *Staphylococcus aureus* (ATCC 29213), *Enterobacter cloacae* (ATCC 13047), *Mycobacterium smegmatis* (ATCC 19420), *Listeria ivanovii* (ATCC 19119), *Streptococcus uberis* (ATCC 29213), and laboratory identified *Vibrio* spp. and *Escherichia coli*.

#### Bacteria culture condition

Antibacterial potential of the essential oil of *T. minuta* flower was tested against four Gram-positive bacteria reference strains which were *S. aureus*, *M. smegmatis*, *S. uberis* and *L. ivanovii* and three Gram-negative bacteria namely *E. cloacae*, *Vibrio* spp. and *E. coli* following CLSI [28] guideline. The bacterial suspensions were made by inoculating a fresh stock culture of the test bacteria strains into tubes containing 5 mL of sterile Luria- Bertani broth and incubated for 24 h at 37 °C. Active cultures grown overnight in sterile Luria- Bertani broth were inoculated into Mueller-Hinton Agar (MHA) incubated for 24 h at 37 °C. After incubation, single colonies were transferred from MHA plates into 4 mL of normal saline solution determined spectrophotometrically at 580 nm as previously reported by Duarte et al., [29] adapted by Omoruyi et al. [24], and the dilutions matching with 0.5 Mc-Farland standard were used for the assay.

#### Determination of antibacterial properties

The modified method of Gullon et al., [30] was adopted for the determination of MIC and MBC of the essential oil*.* Two fold serial dilutions were carried out under aseptic condition in sterile micro centrifuge tubes in a total volume of 100 μL of Muller Hinton (MH) broth mixed with the essential oil of various concentrations ranging from 0.03–0.5 mg/mL. Thereafter, 20 μL of each of the inoculums dilution matching 0.5 Mc-Farland standard was added into tubes of various concentrations and votexed. Dimethyl sulfoxide (DMSO) was used as a diluting chemical solvent as reported by Okoh et al., [31]. Ciprofloxacin (0.03–0.5 mg/mL) was used as positive controls containing the organisms and solution of Ciprofloxacin while DMSO 5% was used as negative control containing only DMSO and the various bacteria strains. The tube containing essential oil and MH broth was used as sterility control. The tubes were incubated at 37 °C for 24 h. MIC was measured by comparing the turbidity of the tubes containing bacteria and essential oil with the tube containing MH broth and essential oil only. MIC was expressed as the lowest concentration without bacteria growth (absence of turbidity). The viability of the bacteria strains screened was verified by inoculating 20 μL of aliquots of all the tubes after incubation of various concentration on MH agar plates using the spread plate method, incubated at 37 °C, for 24 h. MBC was expressed as the lowest concentration of the essential oil that prevented microbial growth on MH agar plates.

#### Statistical analysis

Antioxidant and antibacterial assays were all carried out in triplicate. The results of the essential oil were expressed as standard deviation and correlation coefficients (R^2^) were calculated using Microsoft Excel 2007. IC_50_ values of the essential oil were obtained from the linear regression equation.

## Result

### Physio-chemical characteristics of extracted essential oil of *Tegates minuta* flower

The physio-chemical characteristics of the essential oil are presented in Table 1.

**Table 1:** Physio-chemical characteristics of the essential oil extracted from *T. minuta* flower

Item	Physico-chemical characteristics of EO of *T. minuta* flower
Percentage yield	0.33% *w*/w%
Colour	Very pale yellow
Fragrance (odour)	Pungent odour

The yield of the essential oil was calculated in *w*/w% (per gram) of the extracted plant sample.

### Chemical constituents of the essential oil of *T. minuta* extracted

**Fig. 1:**
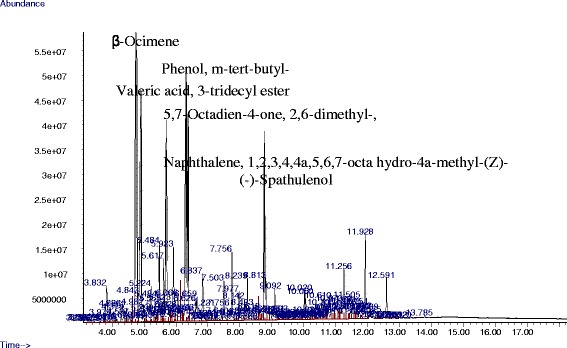
GC-MS spectra of the six major compounds present in the EO of *T. minuta*

The chemical compositions of the essential oil of *T. minuta* flower are shown in Table 2 and the GC-MS analysis of the essential oil of *T. minuta* flower from this study revealed 98 compounds present in the essential oil. The major compounds were β-Ocimene (14.40%), m-tert-butyl-Phenol (9.41%), 2,6-dimethyl-, (E)-5,7-Octadien-4-one(7.14%), 1,2,3,4,4a,5,6,7-octa hydro-4a-methyl-naphthalene (5.58%), and spathulenol (4.56%) as shown in the GC-MS spectra in Fig. 1.

**Table 2:** Chemical composition of the essential oils of *T. minuta* flower

S/N	Chemical constituents	Chemical formula	RT (min)	% Composition
1	Butanoic acid, 2-methyl-, ethyl ester	C_7_H_14_O_2_	3.26	0.06
2	2-Hexenal, (E)-	C_6_H_10_O	3.30	0.09
3	1-Hexanol	C_6_H_14_O	3.37	0.09
4	Heptanal	C_7_H_14_O	3.643	0.05
5	2-Cyclopenten-1-one, 3,4-dimethyl-	C_7_H_10_O	3.752	0.07
6	1H–Imidazole, 2-ethyl-4-methyl-	C_6_H_10_N_2_	3.832	1.29
7	Phenol, 3,4-dimethyl-	C_7_H_10_O	3.923	0.07
8	(1S)-2,6,6-Trimethylbicyclo [3.1.1] hept-2-ene	C_10_H_16_	3.979	0.17
9	Nona-3,5-dien-2-one	C_9_H_14_O	4.022	0.07
10	Camphene	C_10_H_16_	4.16	0.13
11	1-Octene	C_8_H_16_	4.158	0.08
12	Bicyclo [3.1.0] hexane, 4-methylene- 1-(1-methylethyl)-	C_10_H_16_	4.286	0.39
13	Furan, 2-pentyl-	C_9_H_14_O	4.374	0.41
14	Octanal	C_8_H_16_O	4.457	0.36
15	α –Phellandrene	C_10_H_16_	4.537	0.23
16	2,4-Heptanedione, 6-methyl-	C_8_H_14_O_2_	4.648	0.14
17	β-Ocimene	C_10_H_16_	4.751	14.40
18	1,3,7-Octatriene, 3,7-dimethyl-	C_10_H_16_	4.843	0.57
19	Valeric acid, 3-tridecyl ester	C_18_H_36_O_2_	4.903	9.24
20	ɤ-Terpinene	C_10_H_16_	4.982	0.41
21	3,4-Dimethylbenzyl alcohol	C_9_H_12_O	5.039	0.07
22	Furan, 2,3-dihydro-3-methyl-	C_5_H_8_O	5.066	0.15
23	p-Cymen-7-ol	C_10_H_14_O	5.224	1.03
24	6-Methyl-3,5-heptadiene-2-one	C_8_H_12_O	5.299	0.26
25	Furazan, 3-(dimethylaminomethylenamino)-4-(1,2,4-triazol-3-yl)-	C_7_H_9_N_7_O	5.370	0.24
26	2,6-Dimethyl-1,3,5,7-octatetraene, E,E-	C_10_H_14_	5.435	0.07
27	2,4,6-Octatriene, 2,6-dimethyl-,E,Z)-	C_10_H_16_	5.484	1.88
28	5,7-Octadien-4-one, 2,6-dimethyl-, (Z)-	C_10_H_16_O	5.617	1.50
29	5,7-Octadien-4-one, 2,6-dimethyl-, (E)-	C_10_H_16_O	5.698	7.14
30	Sorbic acid vinyl ester	C_8_H_10_O_2_	5.763	0.34
31	Butanoic acid, 3-hexenyl ester, (E)-	C_8_H_18_O_2_	5.923	2.21
32	Bicyclo [2.2.1] heptane, 7,7-dimethy l-2-methylene-	C_10_H_16_	6.028	0.62
33	Decanal	C_10_H_20_O	6.066	0.64
34	Cyclopentanone, 2-cyclopentylidene	C_10_H_14_O	6.121	0.43
35	2-Cyclohexen-1-ol, 2-methyl-5-(1-m ethylethenyl)-, cis	C_10_H_16_O	6.246	0.22
36	Phenol, m-tert-butyl-	C_10_H_14_O	6.318	9.41
37	Naphthalene, 1,2,3,4,4a,5,6,7-octa hydro-4a-methyl-	C_11_H_18_	6.380	5.58
38	Phenol, 2,3,5,6-tetramethyl-	C_10_H_14_O	6.494	0.60
39	4-Methyl-1,3-heptadiene	C_10_H_14_O	6.573	0.26
40	Orcinol	C_7_H_8_O_2_	6.626	0.45
41	1H–Pyrazole, 4,5-dihydro-5,5-dimethyl-4-isopropylidene-	C_8_H_14_N_2_	6.659	0.76
42	Bornyl acetate	C_12_H_20_O	6.750	0.29
43	Propanoic acid, 2-methyl-, octyl ester	C_12_H_14_O_2_	7.032	0.06
44	1,5,5-Trimethyl-6-methylene-cyclohexene	C_10_H_16_	7.128	0.15
45	2,6,10,14-Hexadecatetraen-1-ol, 3, 7,11,15-tetramethyl-, acetate, (E, E,E)-	C_20_H_34_O	7.311	0.06
46	2-Isopropylidene-3-methylhexa-3,5-dienal	C_10_H_14_	7.428	0.18
47	1-Benzothiepin, 2,3,4,5-tetrahydro	C_9_H_10_O_2_S	7.503	1.23
48	Nonyl 2-methylbutanoate	C_14_H_28_O_2_	7.640	0.25
49	Caryophyllene	C_15_H_24_	7.756	1.86
50	Humulene	C_15_H_24_	7.977	0.73
51	Naphthalene, 1,2,3,5,6,7,8,8a–octa hydro-1,8a–dimethyl-7-(1-methylethenyl)-, [1R-(1α,7β,8aα)]-	C_15_H_24_	8.073	0.25
52	Aromandendrene	C_15_H_24_	8.092	0.32
53	1,6-Cyclodecadiene, 1-methyl-5-methylene-8-(1-methylethyl)-, [S-(E,E)]-	C_15_H_26_	8.142	0.51
54	Bicyclogermacrene	C_15_H_24_	8.239	1.19
55	ɤ -Muurolene	C_15_H_24_	8.327	0.17
56	Naphthalene, 1,2,3,5,6,8a–hexahydro-4,7-dimethyl-1-(1-methylethyl)-, (1S–cis)-	C_15_H_24_	8.351	0.15
57	2,2,6-Trimethyl-1-(3-methylbuta-1, 3-dienyl)-7-oxabicyclo [4.1.0] heptan-3-ol	C_14_H_22_	8.423	0.60
58	1,6,10-Dodecatrien-3-ol, 3,7,11-trimethyl-, [S-(Z)]-	C_15_H_24_ O	8.516	0.17
59	Culmorin	C_15_H_24_ O_2_	8.566	0.02
60	Tricyclo[2.2.1.0 (2,6)] heptane, 1,7-dimethyl-7-(4-methyl-3-pentenyl)-, (−)-	C_15_H_24_	8.698	0.19
61	(−)-Spathulenol	C_15_H_24_ O	8.767	4.56
62	Caryophyllene oxide	C_15_H_24_ O	8.813	1.01
63	1H–Cycloprop[e]azulen-4-ol, decahydro-1,1,4,7-tetramethyl-, [1aR-(1aα, 4β, 4aβ,7α, 7aβ, 7bα]-	C_15_H_26_ O	8.865	0.25
64	Levomenol	C_15_H_26_ O	8.914	0.30
65	3-Cyclohexen-1-carboxaldehyde, 3,4-dimethyl-	C_9_H_14_ O	8.965	0.22
66	Tricyclo [4.4.0.0(2,7)] dec-3-ene-3-methanol, 1-methyl-8-(1-methylethyl)-	C_15_H_24_ O	9.092	1.06
67	Longipinocarveol, trans-	C_15_H_24_ O	9.293	0.33
68	Octadecanal	C_18_H_36_ O	9.358	0.11
69	7-Tetracyclo [6.2.1.0(3.8)0(3.9)] undecanol, 4,4,11,11- tetramethyl	C15H24O	9.379	0.11
70	Phenol, 2-methyl-4-(1,1,3,3-tetramethylbutyl)-	C_15_H_24_ O	9.501	0.12
71	Carbonic acid, 4-isopropylphenyl propargyl ester	C_17_H_14_ O_4_	9.551	0.06
72	11-Isopropylidenetricyclo[4.3.1.1(2,5)]undec-3-en-10-one	C_14_H_18_	9.599	0.08
73	Shyobunone	C_15_H_24_ O	9.647	0.03
74	2-Acetyl-6-methoxynaphthalene	C_13_H_12_ O_2_	9.828	0.14
75	Cholesterol	C_27_H_46_ O	9.879	0.04
76	Bicyclo[3.1.1]heptane, 2,6,6-trimethyl-,(1α,2β,5α)	C_10_H_18_	10.020	0.63
77	2-Pentadecanone, 6,10,14-trimethyl	C_18_H_36_ O	10.060	0.53
78	Caryophyllene oxide	C_15_H_24_O	10.124	0.15
79	1-Hexadecanol	C_16_H_34_O	10.230	0.22
80	2,6-Diisopropylnaphthalene	C_16_H_20_	10.300	0.18
81	2-Cyclohexen-1-one, 4-(3-hydroxybutyl)-3,5,5-trimethyl-	C_13_H_22_ O_2_	10.468	0.09
82	6Z-2,5,5,10-Tetramethyl-undeca-2,6,9-trien-8-one	C_15_H_24_ O	10.502	0.09
83	Ar-tumerone	C_15_H_20_ O	10.610	0.50
84	3-(4,8,12-Trimethyltridecyl) furan	C_20_H_36_O	10.706	0.49
85	3,3-Dimethylbutan-2-yl (E)-2-methylbut-2-enoate	C_11_H_20_O_2_	10.920	0.24
86	1-Isopropenyl-3,3-dimethyl-5-(3-methyl-1-oxo-2-butenyl) Cyclopentane	C_15_H_24_O	10.970	1.43
87	3-Methyl-2-butenoic acid, 2-methyloct-5-yn-4-yl ester	C_14_H_22_O_2_	11.022	0.19
88	Fumaric acid, heptadecyl 2,2,2-trifluoroethyl ester	C_8_H_6_F_6_O_4_	11.117	0.40
89	Methyl 3-(1-formyl-3,4-methylenedioxy)benzoate	C_16_H_12_O_5_	11.305	0.42
90	(E)-2-Isopropyl-5-methylphenyl 2-methylbut-2-enoate	Ni	11.523	0.52
91	3-Methyl-2-butenoic acid, 2-methyl	C_14_H_22_O_2_	11.668	0.28
92	Cyclohexane, (2-nitro-2-propenyl)-	C_9_H_15_NO_2_	11.928	2.28
93	Hexane, 1,6-dibromo-	C_6_H_12_Br_2_	11.942	0.33
94	2,2':5',2"-Terthiophene	C_14_H_8_S_3_	12.089	0.25
95	Eicosane	C_20_H_42_	12.175	0.18
96	Benzo[b]naphtho[2,3-d]thiophene,8-dimethyl-	C_18_H_14_	12.591	0.90
97	2,4-Cyclohexadien-1-one, 3,5-bis(1,1-dimethylethyl)-4-hydroxy-	C_14_H_22_O_2_	12.992	0.02
98	Octasiloxane,1,1,3,3,5,5,7,7,9,9,11,11,13,13,15,15-hexadecamethyl-	C_16_H_48_O_7_Si_8_	13.141	0.16
	Total content of EO (%)			89.16
	Yield of EO (%)			0.33

*RT* Retention time, *Ni* not identified, *EO* essential oil

### Essential oil scavenging activity on DPPH radical

**Fig. 2:**
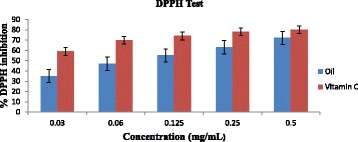
Antioxidant effect of essential oil of *T. minuta* flower and vitamin C on DPPH radical

The essential oil (EO) of *T. minuta* scavenging activity on the DPPH radical is as shown in Fig. 2. The EO as well as the positive control (Vitamin C) displayed concentration-dependent inhibitory effects activities on DPPH radical. The inhibitory effect of the oil at the highest concentration (0.50 mg/mL) is comparable to the inhibitory effect of the vitamin C. The essential oil of *T. minuta* flower displayed highest DPPH inhibitory effect of 72% ± 0.012 at 0.5 mg/mL with IC_50_ value of 2.45 mg/mL while vitamin C displayed higher inhibitory effect (78% ± 0.002) on DPPH radical with IC_50_ value of 0.26 mg/mL.

### Essential oil scavenging activity on ABTS radical

**Fig. 3:**
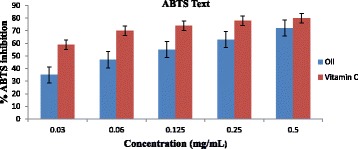
Antioxidant effect of essential oil of *T. minuta* flower and vitamin C on ABTS radical

The percentage ABTS inhibitory effect of the EO of *T. minuta* flower on ABTS radical are shown in Fig. 3. At 0.5 mg/mL concentration of the EO of *T. minuta*, the inhibition was 70% while vitamin C (positive control) was 80%.

### Inhibition of lipid peroxidation

**Fig. 4:**
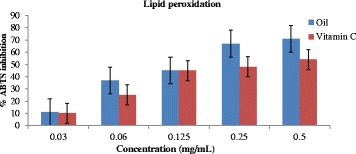
Antioxidant effect of essential oil of *T. minuta* flower and vitamin C on lipid peroxidation radical

The inhibitory action of the essential oil of *T. minuta* flower and vitamin C against lipid peroxyl radical at various concentrations is shown in Fig. 4. The essential oil of *T. minuta* flower percentage inhibition of lipid peroxidation at 0.5 mg/mL was 67% while vitamin C was 54% indicating lower inhibitory scavenging effect compared to the results obtained in DPPH and ABTS tests at same concentration.

### MIC of the essential oil of *T. muinuta* flower

The essential oil of *T. minuta* flower demonstrated good antibacterial activity against all the test bacteria strains. The minimum inhibitory concentration (MIC) value of 0.06 mg/mL was exhibited for *Vibrio* spp., *E. coli*, *E. cloacae* and *L*. *ivanovii*, while the MIC (0.125 mg/mL) for the EO against *S. aurius*, *M*. *smegatis* and *S*. *uberis* was higher as shown in Table 3.

**Table 3:** Minimum inhibitory concentrations (MIC) of essential oil of *T. minuta* flower

Bacteria	Concentrations of essential oil (mg/mL) and MIC (mg/mL)											
	0.5		0.25		0.125		0.06		0.03		MIC(mg/mL)	
Gram positive	A	B	A	B	A	B	A	B	A	B	Oil	Ciprofloxacin
*S. aureus*	−	−	−	−	−	−	+	−	+	−	0.125	0.00
*L. ivanovii*	−	−	−	−	−	−	−	−	+	−	0.06	0.00
*M. smegatis*	−	−	−	−	−	−	+	−	+	+	0.125	0.06
*S. uberis*	−	−	−	−	−	−	+	−	+	−	0.125	0.00
Gram negative												
*E. cloacae*	−	−	−	−	−	−	−	−	+	−	0.06	0.00
*E. coli*	−	−	−	−	−	−	−	+	+	+	0.06	0.125
*Vibro* spp.	−	−	−	−	−	+	−	+	+	+	0.06	0.25

Key: A = Essential oil, B = ciprofloxacin, + = Growth, − = no growth

### MBC of the essential oil

The essential oil of *T. minuta* flower displayed incredible antibacterial activities against Gram-negative bacteria test strains (*E. cloacae, Vibrio* spp. and *E. coli*) and Gram-positive bacteria (*S. uberis*) at varied concentrations as showed in Table 4. The minimum bactericidal concentration of the EO and positive control are shown in Table 4 below.

**Table 4:** Minimum bactericidal concentrations (MBC) of essential oil of *T. minuta* flower

Bacteria	Concentrations of essential oil (mg/mL) and MBC (mg/mL)											
	0.5		0.25		0.125		0.06		0.03		MBC (mg/mL)	
Gram positive	A	B	A	B	A	B	A	B	A	B	Oil	Ciprofloxacin
*S. aureus*	+	−	+	−	+	−	+	−	+	+	0.00	0.06
*L. ivanovii*	+	−	+	−	+	−	+	−	+	−	0.00	0.00
*M. smegatis*	+	−	+	−	+	−	+	−	+	+	0.00	0.06
*S. uberis*	−	−	+	−	+	−	+	−	+	−	0.5	0.00
Gram negative												
*E. cloacae*	−	−	−	−	−	−	−	−	+	−	0.06	0.00
*E. coli*	−	−	−	−	−	−	−	+	+	+	0.06	0.125
*Vibro* spp.	−	−	−	−	−	+	+	+	+	+	0.125	0.25

Key: A = oil, B = ciprofloxacin, + = Growth, − = no growth

## Discussion

The colour and fragrance of the extracted essential oil of *T. minuta* flower obtained in this study were similar to previous report of Wanzala & Ogoma, [32]. Previous study done by Chamorro et al., [33] on the EO from *Tagetes minuta* flower showed that β-Ocimene was reported having the highest chemical content of the EO of *T. minuta* flower and this agrees with our result. However, study done by Shirazi et al., [34] and Garcia et al. [35], reported the predominance of dihydrotagetone in the essential oil of *T. minuta* which was not found in the GC-MS result of the EO of *T. minuta* in our study. Several studies have shown that the chemical composition of the extracted essential oil of *T. minuta* varied according to the location where it was harvested, the growth stage at which it was harvested and the part of the plant used for the extraction [36]. The chemical composition from the GC-MS analysis of the EO of *T. minuta* plant grown in Kenya do not reveal some of the compounds present in our result as reported by Kyarimpa et al. [37], as well as those grown in Argentina [36].

Differences in the chemical constituents of *Tagetes* oil has also been allotted to some environmental variables like soil, temperature and the total period of exposure to sunlight [38]. The differences in the chemical constituents of the EO of *T. minuta* flower could be attributed to many factors which could include but not limited to location, stage of cultivation, season of cultivation and part of the plant used. The chemical composition of the essential oil of *T. mimuta* flower shows various classes of terpenes ranging from hemiterpenes to sesquiterpenes. Similar study done by Wanzala & Ogoma, [32] showed that *T. minuta* essential oil contains a wide range of secondary metabolites mainly sesquiterpenes and monoterpenes including β-ocimene, camphene and bicyclogermacrene and their report corroborates with our result. At 0.5 mg/mL concentration, the EO was 72% while vitamin C was 76% and the EO of *T. minuta* flower displayed lower DPPH radical scavenging activity at various concentrations compared to the vitamin C as shown in Fig. 2. Our result is similar with the report of Muyima et al., [3] on inhibitory effect of the essential oil of *T. minuta* against DPPH radical. The action implicated in antioxidant activity assay is the capacity of the molecule to release hydrogen atom to a radical which is the key factor that is involved in free radical scavenging activity [27, 39]. This effect is displayed as the colour DPPH• fades away (purple to yellow) in the test solution due to the production of neutral DPPH-H molecule upon absorption of hydrogen atom from an antiradical [40]. The sample antioxidant strength is established by the decreased of UV absorption at 517 nm. Study has shown that DPPH method is not an exact radical specie assay but a general radical scavenging strength of an antioxidant compound [41].

For the assumed antioxidant strength of the essential oil of *T. minuta* flower, we used a mono-cation (ABTS radical) and one specific type of specie, the lipid peroxyl radical. The antioxidant activities of the EO of *T. minuta* flower and vitamin C were concentrations dependent as observed in DPPH Test. However, a lower radical scavenging effect of 70% was displayed by EO on ABTS radical at highest concentration (0.50 mg/mL) while vitamin C had higher effect of 80% compared to the DPPH experiment as shown in Fig. 3. The IC_50_ values of 2.76 mg/mL and 1.14 mg/mL for EO flower of *T. minuta* and vitamin C were obtained from the linear regression equation from the graph. Percentage lipid peroxidation inhibitory activity of the EO was higher than that of vitamin C (standard drug) and the inhibitory effects was dose dependent. The essential oil of *T. minuta* flower exhibited the highest lipid peroxidation inhibitory effect of 71% ± 0.001 at the concentration of 0.5 mg/mL with IC_50_ value of 3.23 mg/mL while vitamin C displayed lower percentage lipid peroxidation inhibitory effects of 54% ± 0.004 with IC_50_ value of 4.19 mg/mL as shown in Fig. 4. The result implies that the EO of *T. minuta* flower possess higher lipid peroxidation properties than vitamin C. Antiradical scavenging action of the essential oils may be credited to the substitution of hydroxyl groups of the aromatic ring systems of the phenolic compounds due to their hydrogen giving capacity [42]. Our results in this study demonstrate the ability of the EO of *T. minuta* flower to scavenge three different radicals suggesting it usefulness as a good antioxidant agent for further investigation.

The MIC values of the EO of *T. minuta* against the test bacteria strains as shown in Table 3 displayed that the EO was more active against Gram-negative than Gram-positive bacteria and this report is not in agreement with the report of Senatore et al., [43] and they reported that the MIC value of the EO of *T. minuta* from UK for Gram-positive bacteria were 6.25–25 μg/mL and 25–50 μg/mL for Gram-negative bacteria. Minimum bactericidal concentration of any test sample is the lowest concentration of antimicrobial agents capable of killing or preventing any visible bacteria growth after twenty four hours of incubation under standardized sets of conditions [44, 45]. The EO showed MBC value of 0.06 mg/mL for *E. coli* and *E. cloacae* while at higher concentration of 0.125 mg/mL, it was bactericidal against *Vibrio* spp. and the MBC value for *S*. *uberis* was recorded at 0.5 mg/mL (Table 4). The essential oil of *T. minuta* flower had greater bactericidal effect against *S*. *uberis*, *Vibro* spp. and *E*. *coli*. This suggests that the essential oil of *T*. *minuta* flower may contain some bioactive compounds that could be efficacious in the prevention and treatment of infectious diseases that are linked to these organisms resistant to some antibiotics.

## Conclusion

The results in this present study shows that apart from traditional applications of *T. minuta* plant, the essential oil contained vast bioactive constituents and could serve as a potent resource for new antibacterial and antioxidant agent. However, further studies are required to isolate the main active components, evaluate the bioactivities in-vivo and toxicity of the essential oil of *T. minuta* flower.
